# China’s shifting neglected parasitic infections in an era of economic reform, urbanization, disease control, and the Belt and Road Initiative

**DOI:** 10.1371/journal.pntd.0006946

**Published:** 2019-01-24

**Authors:** Lei Wang, Yang Zou, Xinping Zhu, Maria Elena Bottazzi, Peter J. Hotez, Bin Zhan

**Affiliations:** 1 Beijing Institute of Tropical Medicine, Beijing Friendship Hospital, Capital Medical University, Beijing, PR China; 2 Departments of Pediatrics and Molecular Virology and Microbiology, National School of Tropical Medicine, Baylor College of Medicine, Houston, Texas, United States of America; 3 Beijing Key Laboratory for Prevention and Treatment of Tropical Diseases, Beijing, PR China; 4 Department of Medical Microbiology and Parasitology, School of Basic Medical Sciences, Capital Medical University, Beijing, PR China; 5 Department of Biology, Baylor University, Waco, Texas, United States of America; Liverpool School of Tropical Medicine, UNITED KINGDOM

## A shifting pattern of parasitic diseases in China

Published estimates from China’s Ministry of Health indicated that by the early 1990s China exhibited some of the world’s highest prevalence rates of parasitic and other tropical diseases [1]. The findings included estimates that more than 0.5 billion people were infected with ascariasis, whereas approximately 200 million people suffered from trichuriasis and hookworm infection [2, 3]. In the more than two decades since the first published estimates of China’s parasitic infections, the nation has undergone impressive economic development, with its GDP growth roughly increased by 10% annually and massive reductions in the numbers of Chinese living in extreme poverty [4]. Indeed, the World Bank notes that China was successful in achieving all of its Millennium Development Goals [4]. As a result, today China is the second largest economy globally next to the United States.

**Fig 1:**
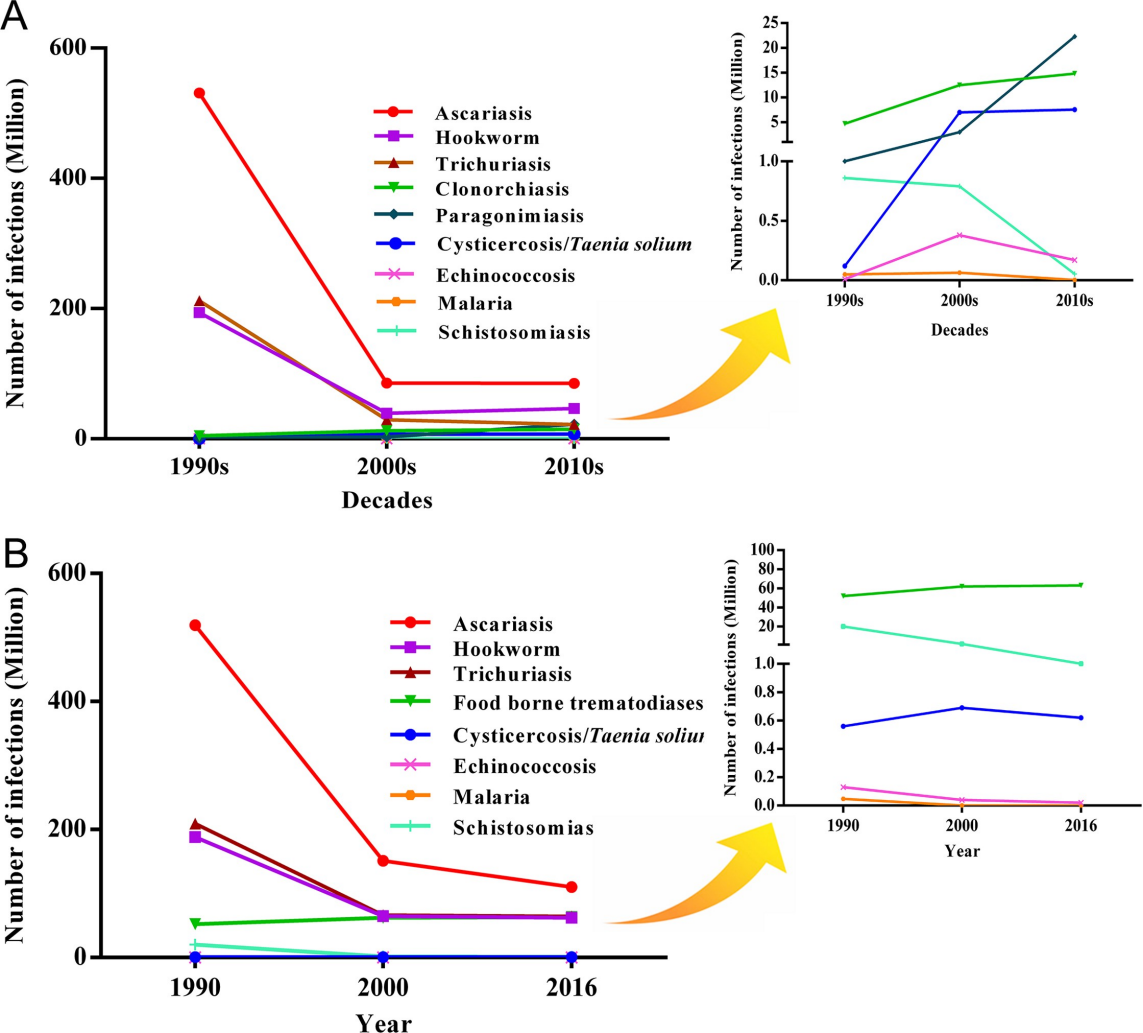
**The overall trends of China’s major neglected parasitic infections since 1990 based on information either from (A) several national surveys performed by Ministry of Public Health and other sources [1, 2, 6, 7] or (B) the GBD 2016 [8].** The second graphs on the right are the amplified graphs for those parasitic diseases with low prevalence on the bottom. GBD, Global Burden of Disease.

In addition to rapid economic growth that promotes urbanization, significant change in agricultural practice, and improved sanitation, China has undertaken extensive parasite control measures [5]. These changes have translated into impressive reductions of China’s neglected parasitic diseases and other neglected tropical diseases (NTDs) during recent decades. Based on several national surveys for parasitic diseases performed by Ministry of Public Health and other public health agencies in 1994 [1, 2], 2005 [6], and 2010 [7], as well as the data from Global Burden of Disease (GBD) 2016 [8], the overall trends of China’s major neglected parasitic infections are shown in Fig 1.

Overall there is general concurrence between both referenced data sets. Briefly, China’s major neglected parasitic diseases have declined dramatically, especially soil-transmitted helminth infections (STHs), schistosomiasis [5], and malaria [9], and lymphatic filariasis has been eliminated [10]. However, the prevalence of some food-borne trematode infections and cysticercosis has significantly increased [11]. Although autochthonous malaria has been nearly eliminated in China, imported malaria and other parasitic infections have been brought into China from Africa or other endemic countries as trade activities and traveling have increased during the past decades, especially after the newly launched Belt and Road Initiative.

### Declining of endemic STHs and schistosomiasis

The major STHs—ascariasis, trichuriasis, and hookworm infection—have been historically widespread in China and have had a major role in interfering with economic and social advancement [12]. Based on a nationwide survey of human parasites in China conducted in total 1,477,742 persons in 1994, the prevalence rate of ascariasis, hookworm infections, and trichuriasis was 47.0%, 17.2%, and 18.8%, with estimates of a total infected population of 531 million, 194 million, and 212 million, respectively [1, 13]. However, China’s economic growth has not occurred evenly with an East to West poverty gradient, resulting in dramatic declines in the former and remaining high STHs prevalence rates in the southwest provinces where the economy has not progressed [14]. For example, recent estimates indicate that the highest prevalence rates of STHs currently occur in the southwestern provinces of Guangxi, Guizhou, Sichuan, and Yunnan, where poverty is widespread, as well as the South China Sea Province of Hainan [7].

Schistosomiasis caused by the infection of *Schistosoma japonicum* has had an important role in the history of modern China. Prior to the Great Leap Forward in the 1950s, it was highly endemic in 12 provinces of the Yangtze River valley, with 11.6 million infected individuals and over 100 million people under threat of infection [15, 16]. Since then, China has implemented comprehensive national campaigns to control the prevalence of schistosomiasis in humans and livestock, through the liberal use of molluscicides and destroying snail habitats, mechanization to replace water buffalo with tractors, and large-scale mass chemotherapy of both humans and livestock with praziquantel [17]. According to the Ministry of Health, the number of infected human cases has dropped to 54,454 in 2016 with no new acute cases reported [18]. However, these estimates are significantly lower than the GDB 2016, which reported schistosomiasis prevalence of 1,067,203 in 2016 in China, possibly due to the different cited sources [8]. We are working to understand the basis of this discrepancy. There are also concerns about snail repopulation along the Yangtze and its tributaries, and human disease reemergence as a consequence of climate change and large hydroelectric projects such as the Three Gorges Project and the South-to-North Water Diversion Project in China [19, 20].

### Malaria

**Fig 2:**
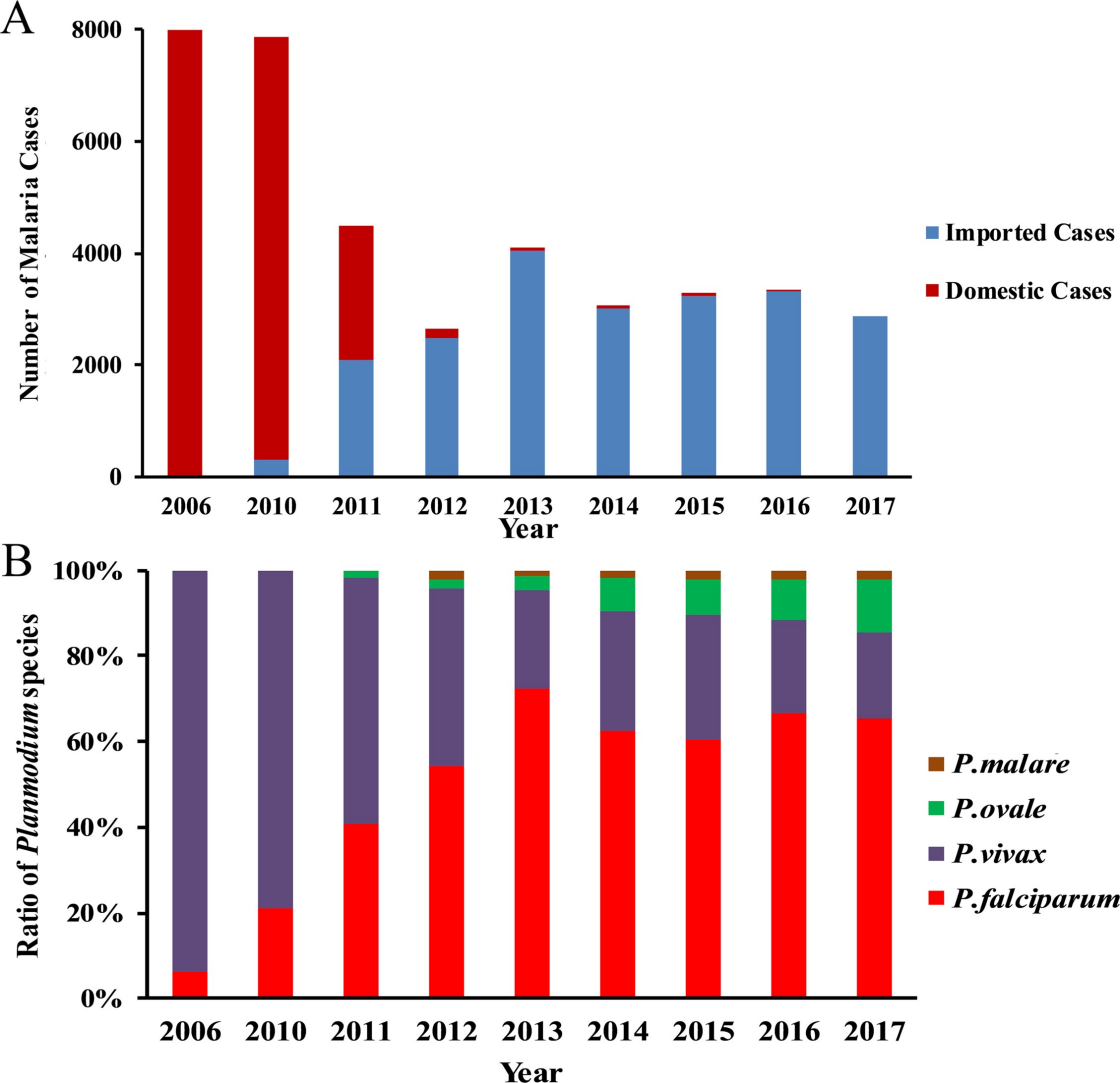
The dynamic changes of malaria prevalence in China since 2006 [21–24, 53–57]. (A) Imported and Domestic malaria cases presented in different years. (B) Infected *Plasmodium* species reported in China since 2006.

China’s indigenous malaria, mostly caused by *Plasmodium vivax*, was effectively controled after several decades of nationwide efforts, which included the screening and treatment of patients, integrated vector control, and a nationwide surveillance and reporting systems [9]. As a result, outstanding progress has been made toward the elimination of malaria in China. Since the National Malaria Elimination Program was launched in 2010, China’s malaria incidence has declined from 64,178 cases in 2006 [21] to 2,718 cases in 2012 [22] and has remained at low levels (Fig 2A). However, there has been a significant rise in imported cases from Africa and southeast Asia, especially since the launch of Belt and Road Initiative. In 2016 alone, there were 3,321 malaria cases reported; 3,317 of them were imported from Africa or other endemic countries with only 3 cases of indigenous malaria [23]. There was no indigenous malaria case reported in China in 2017; all of the malaria cases were imported [24]. This is associated with a shift in malaria species from predominantly *P*. *vivax* to *P*. *falciparum* in addition to *P*. *ovale* and *P*. *malariae* (Fig 2B) [24]. Moreover, although the total cases of malaria have been significantly reduced, the severe malaria cases and deaths remain at similar levels due to the increased proportion of imported *P*. *falciparum* malaria [16, 23].

### Increasing food-borne helminth infections

The prevalence of foodborne parasitic diseases has risen sharply during the last two decades, such that these diseases have emerged as important illnesses affecting food safety and public health in both urban and rural foci [11]. Paradoxically, the rise in China’s foodborne parasitic infections may partly reflect rising incomes, with resultant increased access to meat or exotic foods, together with urbanization [25].

Clonorchiasis and paragonomiasis represent two key food-borne trematode infections in China. With regard to the former, approximately 15 million people are infected nationwide [11, 26], representing an 80% increase compared with the infections identified in the first national survey of parasitic diseases in 1994 [2]. Today, China accounts for 85% of global clonorchiasis. The geographical distribution shows a north-south polarized distribution with the highest infection rates in the southern provinces of Guangdong and Guangxi, or in the northern provinces of Heilongjiang and Jilin [26]. Due to its identification as a strong carcinogen, infection of *Clonorchis sinensis* has become an important cause for liver cancer in these endemic areas [11]. With regards to paragonimiasis, the current national infection rate is 1.7%, with an estimated infected population of 22.3 million with 200,000 disability-adjusted life year (DALY) lost[6, 25, 27].

Two important food-borne zoonoses from pigs include trichinellosis and taeniasis-cysticercosis. For trichinellosis, the infected population is estimated over 20 million, with 40 million people at risk in China, and more than 2 billion Chinese yuan (CNY) spent on inspection and quarantine of pigs annually [25, 28]. Taeniasis-cysticercosis caused by *Taenia solium* is also widespread. The prevalence of cysticercosis in China increased from 0.01% in 1994 to 0.58% in 2004, so that the most recent estimate indicates that the infected population was about 7 million [6]. Tibet in Western China exhibits the highest *Taenia* infection rate of 19.2% [6, 29]. Echinococcosis is also endemic in western China, with an estimated 170,000 infections, of which more than 98% occur in Xinjiang, Qinghai, Gansu, Sichuan, Inner Mongolia, and Ningxia [30].

### Belt and Road Initiative and imported parasitic diseases

**Fig 3:**
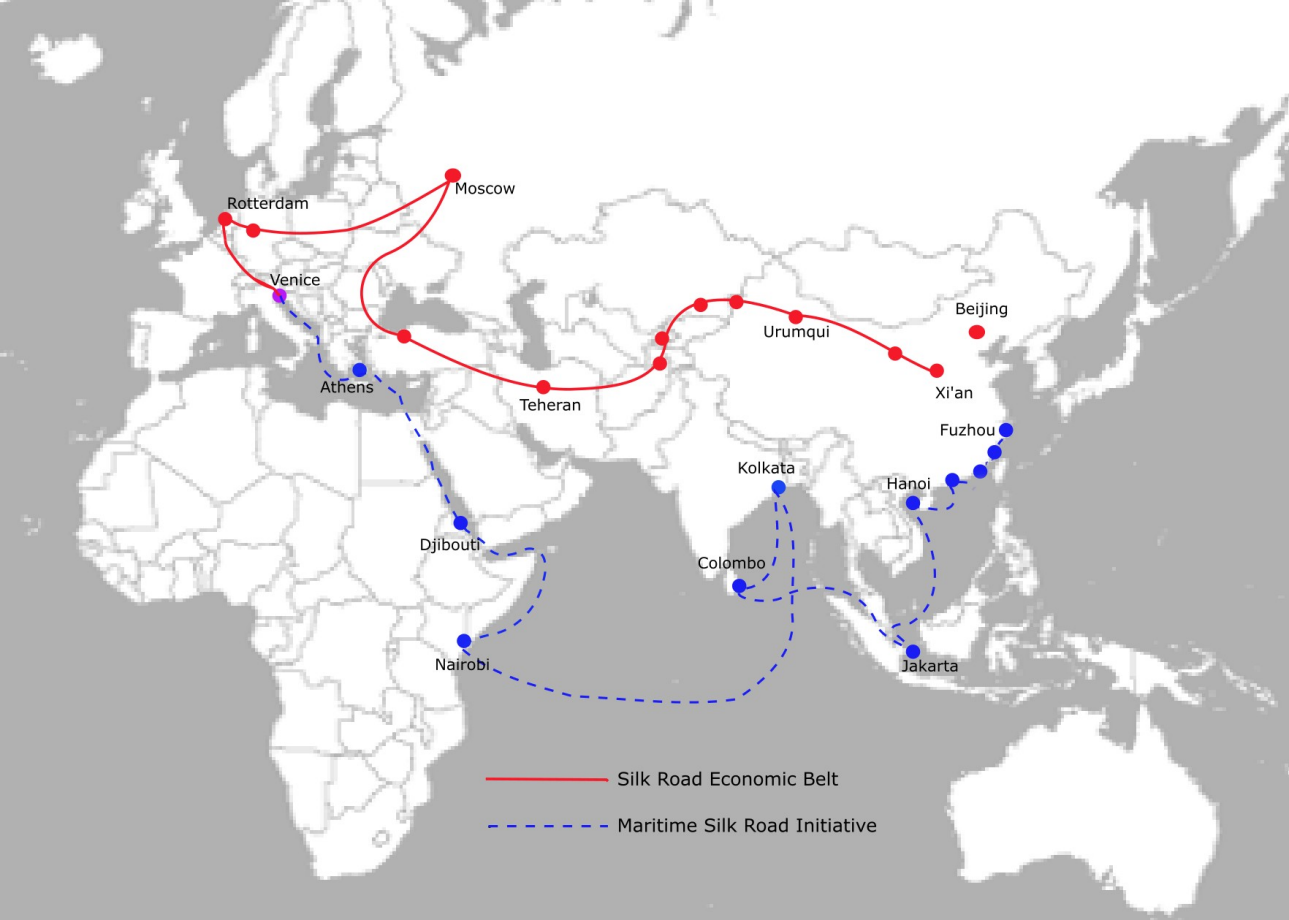
Map of China’s proposed Belt and Road Initiative including a land route (One Road; red unbroken line) and a sea route (One Belt; blue dotted line) reaching from China to Asia, Europe, and Africa. Original figure made with data from China Xin Hua news agency and public domain world map available here: https://commons.wikimedia.org/wiki/File:BlankMap-FlatWorld6.svg [52].

In 2013, Chinese President Xi Jinping launched the landmark Belt and Road Initiative to enhance trade, infrastructure, and economic outreach from China to and from Asia, Africa, and Europe [31]. The new initiative is being touted as one of the most ambitious economic and foreign policy initiatives undertaken by China since its liberation in 1949 (Fig 3).

With respect to Africa, currently there are an estimated 3 million Chinese already working there through more than USD$100 billion in capital investments made at the World Economic Forum in Davos [32]. In terms of emerging parasitic diseases, however, a significant downside of increased investments and trade in Africa has been an increase in imported parasitic infections including intestinal schistosomiasis (caused by *Schistosoma mansoni*), loiasis, African trypanosomiasis, cutaneous leishmaniasis [33–37], and *falciparum* malaria.

## Strategy to effectively control the neglected parasitic diseases in China

As highlighted above, there are three major themes to China’s neglected parasitic infections: (1) as nationwide control performed in China, STHs and schistosomiasis have significantly declined, however, STH infections still remain highly prevalent in China’s southwest due to persistent poverty; (2) increases in food-borne helminth infections in northern and southern provinces; and (3) imported tropical infections, led by *falciparum* malaria. In addition, China is becoming increasingly urbanized with population shifts leading to the establishment of new megacities—by some estimates, 60% of China’s population will live in urban areas, with the creation of multiple megacities by the year 2030 [38]. By this time, one-quarter of the world’s 100 largest cities will be in China. Unchecked urbanization has the capacity to promote the emergence of urban STHs and schistosomiasis, malaria, and leishmaniasis[39]. Based on these trends of parasitic infections in China, the following strategies should be considered to strengthen the control of these infections.

### Comprehensive control measures

For the future control of STHs, there is an urgent need to continue implementing mass drug administration approaches using albendazole and mebendazole, especially to China’s less-developed southwestern provinces and Hainan. However, high rates of post-treatment reinfection and variable efficacies of benzimidazole anthelminthics, including the possible emergence of anthelminthic resistance [40, 41], suggest the need for alternative technologies, including better drugs or even anthelminthic vaccines [42]. For schistosomiasis in China, enormous strides have been made through a multipronged approach highlighted above, but there is a need to reinforce and strengthen these measures to achieve its elimination as a public health problem. For food-borne helminth infections, a comprehensive control approach should be deployed, for example, the establishment of case report network, including a traceable system for the infection source; a surveillance and supervision system for the entire food industry chain; and public education and awareness for disease infection and control methods [43, 44].

### International monitoring and surveillance network

For the importation of malaria and other NTDs, there is a need to expand monitoring and disease surveillance, especially among the estimated 3 million Chinese workers in Africa and the more than 430,000 Africans living in Guangdong province for academic study, business, and trade [45]. In parallel, there is a need to establish tropical disease control and surveillance centers in the major urban areas of China, such as the one established at the Friendship Hospital in Beijing [34], which plays important roles in screening, diagnosis, and treatment of imported tropical diseases. Conversely, within Africa, China’s national aid can be partly redirected to the healthcare sector with an emphasis on NTD surveillance and treatments. Currently, the healthcare sector accounts for only 2.1% of China’s total investment in Africa, and only a small percentage of this amount goes towards basic and clinical research and training [46, 47]. Expansions in public health and medical research support for African countries would promote both disease reductions there and in imported tropical diseases to China.

The Belt and Road Initiative also has implications beyond Africa [48]. In the Middle East, a wide range of NTDs are emerging in the conflict zones of Syria, Iraq, Libya, and Yemen, which will be further disseminated through roadmapped trade routes. Shown in Table 1 are some of the major NTDs we can anticipate emerging in China as a consequence of Belt and Road trade [48, 49].

**Table 1:** Major NTDs and malaria in countries along the Belt and Road Initiative.

Diseases	East Africa	South-Southeast Asia	Middle East	Russia-Kazakhstan-Central Asia	West China (One Road start)	East China (One Belt start)
Malaria	√	√				
Visceral leishmaniasis	√	√		√	√	
Cutaneous leishmaniasis			√	√	√	
Schistosomiasis	√		√			√
Food-borne helminthiasis				√		√
Echinococcosis			√	√	√	
STHs	√	√				√
MERS			√			
Dengue and arbovirus		√				√

**Abbreviations:** MERS, middle east respiratory syndrome; NTD, neglected tropical disease; STH, soil-transmitted helminth infection.

## Concluding comments

**Fig 4:**
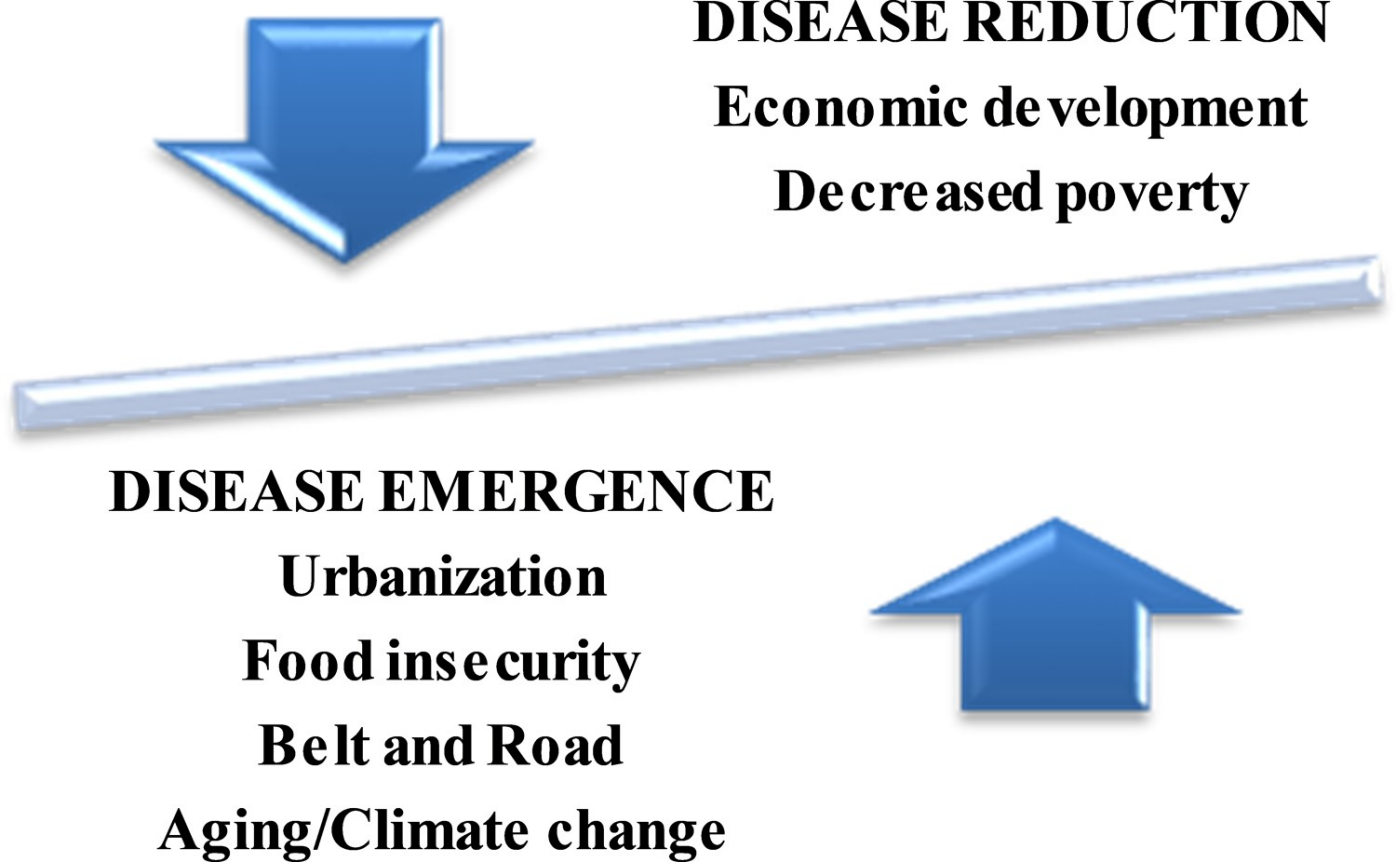
Dynamic forces that affect China’s neglected tropical diseases (NTDs).

Shown in Fig 4 are some of the modern 21st century forces that are likely to affect the future of China’s human parasitic infections. They include continued disease prevalence reductions due to further decrease in poverty (especially rural poverty) but also new factors that could promote disease emergence, including the rise of urbanized helminth infections as noted elsewhere; continued food insecurity, especially in terms of its impact on food-borne trematodiases and other helminth infections; and China’s Belt and Road initiative in terms of its reintroduction of malaria and other tropical infections. Finally, although not discussed in detail here, we’ll need to consider the impact of China’s aging population on parasitic infections. For instance, it was noted that the elderly are disproportionately affected by hookworm infection in some areas of China [50]. Also, more attention needs to be paid to the possible effects of climate change on the emerging or reemerging NTDs, especially on vector-borne and snail-borne disease [51]. It will be important to continue active surveillance for parasitic infections in order to better understand the dynamic state of China’s human parasitic infections.
